# Opportunities for enhanced surveillance of foot‐and‐mouth disease in endemic settings using milk samples

**DOI:** 10.1111/tbed.13146

**Published:** 2019-02-27

**Authors:** Bryony Armson, Jemma Wadsworth, Tito Kibona, Deogratius Mshanga, Veronica L. Fowler, Nick J. Knowles, Valérie Mioulet, Richard Reeve, Donald P. King, Katarzyna Bachanek‐Bankowska, Tiziana Lembo

**Affiliations:** ^1^ The Pirbright Institute Pirbright Surrey UK; ^2^ Boyd Orr Centre for Population and Ecosystem Health Institute of Biodiversity, Animal Health and Comparative Medicine College of Medical, Veterinary and Life Sciences University of Glasgow Glasgow UK; ^3^ Sokoine University of Agriculture Morogoro Tanzania; ^4^ Nelson Mandela African Institution of Science and Technology Arusha Tanzania; ^5^ Tanzania Veterinary Laboratory Agency Ministry of Livestock and Fisheries Arusha Tanzania

**Keywords:** East‐Africa, foot‐and‐mouth disease virus, milk, real‐time RT‐PCR, surveillance, Tanzania

## Abstract

Under‐reporting of foot‐and‐mouth disease (FMD) masks the true prevalence in parts of the world where the disease is endemic. Laboratory testing for the detection of FMD virus (FMDV) is usually reliant upon the collection of vesicular epithelium and fluid samples that can only be collected from acutely infected animals, and therefore animals with sub‐clinical infection may not be identified. Milk is a non‐invasive sample type routinely collected from dairy farms that has been utilized for surveillance of a number of other diseases. The aim of this study was to examine the application of milk as an alternative sample type for FMDV detection and typing, and to evaluate milk as a novel approach for targeted surveillance of FMD in East Africa. FMDV RNA was detected in 73/190 (38%) individual milk samples collected from naturally infected cattle in northern Tanzania. Furthermore, typing information by lineage‐specific rRT‐PCR assays was obtained for 58% of positive samples, and corresponded with the virus types identified during outbreak investigations in the study area. The VP1‐coding sequence data obtained from milk samples corresponded with the sequence data generated from paired epithelial samples collected from the same animal. This study demonstrates that milk represents a potentially valuable sample type for FMDV surveillance and might be used to overcome some of the existing biases of traditional surveillance methods. However, it is recommended that care is taken during sample collection and testing to minimize the likelihood of cross‐contamination. Such approaches could strengthen FMDV surveillance capabilities in East Africa, both at the individual animal and herd level.

Foot‐and‐mouth disease (FMD) is a highly contagious disease of cloven‐hooved mammals and is of great global economic importance (Knight‐Jones & Rushton, 2013). There are seven serotypes of FMD virus (FMDV), O, A, C, Asia 1 and Southern African Territories (SAT) 1, SAT 2 and SAT 3 (Robson, Harris, & Brown, 1977), four of which (O, A, SAT 1 and SAT 2) currently circulate in domestic livestock in East Africa, where the disease is endemic. Vaccination is the most effective control measure for FMD prevention. In order to identify the appropriate vaccines and the time of their application, a thorough understanding of contemporary serotypes/strains is necessary (Casey‐Bryars et al., 2018; Kasanga et al., 2012; Paton, Sumption, & Charleston, 2009). However, rapid viral detection and characterization can often be problematic in endemic areas due to limited resources and capacity to undertake surveillance.

Pan‐serotypic real‐time reverse‐transcription polymerase chain reaction (rRT‐PCR) assays have been described for the rapid detection of FMDV in typical clinical samples (Callahan et al., 2002; King et al., 2006; Reid et al., 2002; Shaw et al., 2007). These assays target highly conserved genomic regions that are shared among all serotypes and topotypes, but do not differentiate between them. To enable rapid typing of FMDV, rRT‐PCR assays have also been developed for the detection and differentiation between FMDV lineages specific to particular geographical regions, including East Africa (Bachanek‐Bankowska et al., 2016). These region‐tailored typing assays target lineage‐specific conserved regions within the variable VP1‐coding sequence (Bachanek‐Bankowska et al., 2016; Reid et al., 2014; Saduakassova et al., 2017).

Surveillance for FMD in an endemic setting such as East Africa often relies on passive surveillance, depending on farmers or veterinarians to observe and report infected herds, and is only rarely supplemented by targeted case finding (Kasanga et al., 2012; Namatovu et al., 2013). Furthermore, sample types for laboratory FMDV diagnosis, typically vesicular epithelium and fluid, are invasive and labour intensive to obtain, and therefore, they are collected infrequently, resulting in under‐reporting (Knight‐Jones, McLaws, & Rushton, 2016). As a consequence, FMD reporting is inherently biased towards clinically affected animals, failing to capture viruses circulating sub‐clinically that may play a role in disease transmission.

Milk is routinely collected from dairy farms, and has been exploited as a surveillance tool for the detection of other diseases of veterinary importance, for example bovine viral diarrhoea, border disease and bluetongue (Beaudeau et al., 2001; Berriatua et al., 2006; Kramps, van Maanen, Mars, Popma, & van Rijn, 2008). It has been demonstrated that the mammary gland is a highly susceptible organ for FMDV replication, and that during infection, FMDV RNA can be detected in milk by rRT‐PCR before, during and after the appearance of clinical signs (Armson et al., 2018; Blackwell & McKercher, 1982; Burrows, Mann, Greig, Chapman, & , 1971; Reid et al., 2006). However, only a small number of studies have described the detection of FMDV RNA in milk from naturally‐infected animals. These include FMDV detection in milk during the 2007 FMD outbreak in the United Kingdom (Armson et al., 2018), in cattle and buffaloes in Pakistan (Ahmed et al., 2017; Saeed et al., 2011) and in cattle in India (Ranjan et al., 2016). The limited milk samples used in these studies were collected either as an additional sample type to validate molecular assays, or to investigate the possible role of milk in FMDV transmission; nonetheless these studies provide useful evidence that FMDV RNA can be detected and typed by rRT‐PCR, in milk from naturally infected animals. Consequently, further investigation into the potential of milk as an alternative non‐invasive sample type for routine FMDV detection and surveillance is warranted, particularly in areas where surveillance infrastructure is limited. Therefore, the aim of this study was to examine the use of milk for surveillance in endemic settings of East Africa where this approach had not been investigated to date.

For this study, milk samples (*n* = 190) were collected by hand from clinical and healthy cows during FMD outbreak investigations in northern Tanzania (Serengeti and Bunda Districts) between 2012 and 2015 (Casey‐Bryars et al., 2018). For four of the FMD clinically affected cows that supplied a milk sample, vesicular lesion material (epithelium or fluid) was also collected on the same day, and samples tested in duplicate. This lesion material was submitted to the FAO World Reference Laboratory for FMD (WRLFMD; The Pirbright Institute, UK) for confirmatory diagnostics, sequencing and phylogenetic analyses (WRLFMD, 2015) (Table S1).

An initial screen of all the milk samples and the lesion material from four of the cows was performed. For this, RNA was extracted using the MagMAX^™^‐96 Viral RNA Isolation Kit (Applied Biosystems^®^). The OIE recommended pan‐serotypic rRT‐PCR assay was carried out on an Applied Biosystems^®^ 7500 Real‐time PCR System, using the Superscript III Platinum^®^ One‐Step qRT‐PCR Kit (Invitrogen^™^), with primers and probes targeting the conserved three‐dimensional region of the FMDV genome (Callahan et al., 2002; OIE Terrestrial Manual, 2017), and thermal cycling conditions as previously reported (Shaw et al., 2007). Positive samples were then tested using East Africa (EA) typing rRT‐PCR assays, as previously described (Bachanek‐Bankowska et al., 2016). RNA extracted from cell culture isolates TAN/39/2012, TAN/6/2013, TAN/33/2014 and TAN/19/2012 (Table S1) supplied by the WRLFMD were used as rRT‐PCR assay positive controls. For all rRT‐PCR assays, positive samples were defined as those with a *C*_T_ ≤ 50.

FMDV RNA was detected in 73/190 (38%) of milk samples (Figure 1a) and the FMDV type was identified in 42/73 (58%) of FMDV positive milk samples (Figure 1b). SAT 1 was the most prevalent serotype detected (45%), followed by serotypes O (29%) and A (12%), with no evidence of SAT 2 in the milk samples tested (Figure 1b, Table S1). Milk samples that were observed to have a *C*_T_ value of above 38 using the pan‐serotypic rRT‐PCR assay were unable to be typed. In addition, a positive signal from more than one typing assay was identified in 18 milk samples, including three samples each positive for three serotypes (O, A and SAT 1). In samples with a positive signal for two FMDV types, O and SAT 1 were the most common types detected, while types A and SAT 1 were identified in one sample only. It is possible that these animals were co‐infected with multiple FMDV serotypes, as has been previously described in endemic areas (Ferris, Oxtoby, & Hughes, 1995; Woodbury, Samuel, Knowles, Hafez, & Kitching, 1994). However, alternative explanations should also be considered, including the possibility that these results represent (a) contamination due to contact with materials infected with other FMDV types during sample collection in the field, transport or testing in the laboratory; or (b) cross‐reaction between the individual typing rRT‐PCR assays, although no evidence of this has been observed during the validation of these tests (Bachanek‐Bankowska et al., 2016).

**Figure 1:**
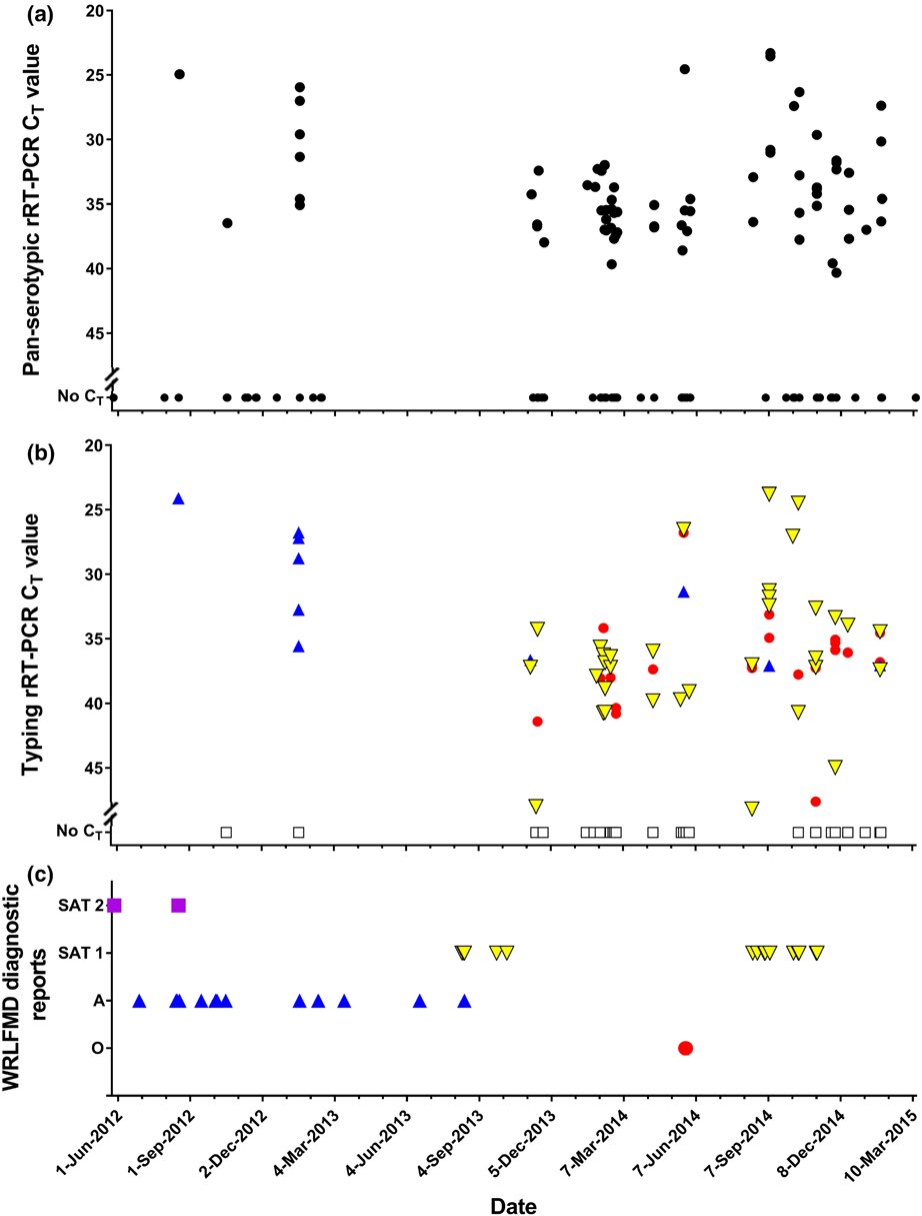
(a) *C*_T_ values from the pan‐serotypic real‐time reverse‐transcription polymerase chain reaction (rRT‐PCR) assay (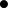) for milk samples collected from individual cows in northern Tanzania throughout the study period (*n* = 190). (b) *C*_T_ values for each East African serotyping rRT‐PCR assay for samples that tested positive (*C*_T_ ≤ 50) in the pan‐serotypic rRT‐PCR assay. 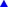: Serotype A. 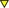: Serotype SAT 1. 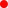: Serotype O. 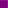: Serotype SAT 2. 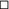: Sample that could not be typed. (c) Collection dates and the reported serotypes of clinical samples (vesicular epithelium/fluid) submitted to the World Reference Laboratory for Foot‐and‐mouth disease (WRLFMD) [Colour figure can be viewed at wileyonlinelibrary.com]

Published reports of clinical samples from the study region indicate circulation of all four serotypes during the study period (WRLFMD, 2015; Figure 1c) and are mainly consistent with rRT‐PCR typing results of the milk samples. Although serotype SAT 2 was identified in clinical samples collected in Northern Tanzania at the start of the study period, milk samples were not collected from these specific locations, which may explain the absence of serotype SAT 2 in the milk samples. On some dates, FMDV RNA was detected in a milk sample, but there were no confirmed diagnostic reports of this serotype in the region at this time. This could be due to poor farmer recognition of clinical signs, lack of disease reporting, or sample contamination (as discussed above). Alternatively, these results may indicate that FMDV can be detected in milk samples during the pre‐clinical or convalescence phases of infection, as reported previously (Armson et al., 2018; Blackwell & McKercher, 1982; Reid et al., 2006), or even during subclinical infection.

To determine if milk is a suitable alternative sample type to vesicular lesion material (epithelium/fluid) for FMDV detection and typing, both sample types collected from the same animal were tested and the results compared (Table 1). In the pan‐serotypic assays, the *C*_T_ values of the lesion material samples were stronger (lower *C*_T_ values) than of the milk samples, confirming previous observations of higher virus concentrations in vesicular lesions (King et al., 2006). Typing results were comparable for all pairs, with the exception of Animal A, where no signal was observed in any of the typing assays, possibly due to the reduced viral load observed in this animal. In three animals (B, C and D), SAT 1 was detected in both milk and lesion material samples (Table 1). In animals C and D, the *C*_T_ values of the pan‐serotypic and the SAT 1‐specific assays were comparable, while in animals A and B the differences in the values were greater. In animal A, SAT 1 was detected in the vesicular lesion sample only. Two different FMDV types (O and SAT 1) were detected in both milk and vesicular fluid in animal B, but type O was not detected in the vesicular epithelium sample. As discussed above, contamination cannot be excluded as a reason for these results. Observations from paired samples indicate that, despite a weaker rRT‐PCR signal, milk can be used for detection and typing of FMDV in most individual samples.

**Table 1:** FMDV detection in milk samples and epithelial samples

Animal reference	WRLFMD reference/milk sample	Sample type	3D	O	A	SAT 1	SAT 2
A	TAN/20/2014	Vesicular epithelium	18.30	No *C*_T_	No *C*_T_	24.98	No *C*_T_
	7736	Milk	33.31	No *C*_T_	No *C*_T_	No *C*_T_	No *C*_T_
B	TAN/22/2014	Vesicular epithelium	10.19	No *C*_T_	No *C*_T_	21.06	No *C*_T_
	TAN/23/2014	Vesicular fluid	9.94	38.07	No *C*_T_	19.76	No *C*_T_
	7609	Milk	29.04	33.13	No *C*_T_	33.42	No *C*_T_
	TAN/28/2014	Vesicular epithelium	16.00	No *C*_T_	No *C*_T_	20.33	No *C*_T_
C	TAN/29/2014	Vesicular fluid	9.05	No *C*_T_	No *C*_T_	10.3	No *C*_T_
	7805	Milk	26.48	No *C*_T_	No *C*_T_	29.17	NP
D	TAN/34/2014	Vesicular epithelium	16.51	No *C*_T_	No *C*_T_	16.77	No *C*_T_
	7815	Milk	25.08	No *C*_T_	No *C*_T_	25.69	NP

*Note*

*C*_T_ values are the mean of duplicates for each rRT‐PCR assay. NP: not performed.

VP1 sequences obtained from milk samples 7805 (animal C; accession number MH791039) and 7815 (animal D; accession number MH791040) were found to be identical (animal D) or within one nucleotide difference (animal C) to reported sequences of paired vesicular samples from the same animals (animal C: accession number MF592687, animal D: accession number MF592691) (data not shown). The nucleotide difference for animal C was a non‐synonymous change at VP1 amino acid position 204. This nucleotide difference may be explained by a mutation that could have occurred during viral replication, as sequences from the vesicular samples were obtained from virus isolated on primary bovine thyroid (BTY) cells. Upon comparison of the SAT 1‐specific primers/probe with the VP1‐coding sequence data obtained from milk and vesicular samples, it was evident that the difference in *C*_T_ values between the pan‐serotypic and the SAT 1‐type specific assay may occur due to nucleotide differences at the 3’ end of the primer binding region of the typing assay. At least one nucleotide difference was identified within the SAT 1‐specific typing assay binding region in sequences obtained from animals A and B, while no such differences were observed in sequence data obtained from animals C and D. As the VP1‐coding sequence is the most variable genome region, mismatches between the primers and probes of the typing assays and the template are expected. Therefore, it is recommended to use typing assays alongside the more sensitive pan‐serotypic assay as a screening tool (Bachanek‐Bankowska et al., 2016).

This study demonstrates that milk could represent a valuable sample type as an alternative to the traditional diagnostic samples collected for FMD surveillance: vesicular epithelium or fluid. Milk from individual animals can be routinely collected and FMDV RNA can be detected and typed by rRT‐PCR in milk samples in a region where FMD is endemic, albeit with weaker *C*_T_ values than from vesicular samples. The study also demonstrates that VP1 sequence data may be obtained from milk samples, enhancing the possibility of further, in‐depth virus characterization. Milk sampling as a targeted surveillance approach shows promise given the concordance between typing data from milk samples and confirmed reports from outbreak investigations. Follow‐on studies are required to assess the application of pooled milk in combination with herd clinical status for FMDV surveillance. In conclusion, milk is a simple‐to‐collect, non‐invasive sample type which might be utilized in targeted surveillance campaigns in FMD endemic regions. However, due to the high analytical sensitivity of molecular tests used to detect FMDV, appropriate care needs to be taken to minimize the possibility for cross‐contamination during sample collection, transport and testing in the laboratory. The use of milk as a diagnostic sample might help to address some of the potential biases of traditional surveillance methods and improve surveillance capabilities for reporting of disease at the individual and herd level in East Africa.

## CONFLICT OF INTEREST

The authors have no competing interests.
