# Supplementary material for: Opportunities for enhanced surveillance of foot‐and‐mouth disease in endemic settings using milk samples
Source: Transbound Emerg Dis. 2019 Feb 27;66(3):1405–10. doi: 10.1111/tbed.13146 (PMC6563157; doi:10.1111/tbed.13146)
Supplement: Supplementary file 1 [file TBED-66-1405-s001.docx]

**Supplementary Data**

Supplementary Data 1. List of samples and virus isolates from northern Tanzania used throughout the study. The mean C_T_ values for the pan-serotypic and East Africa (EA) typing assays are reported.

| **Animal ID/ WRLFMD Reference** | **Collection date** | **Sample type** | **Location** |  | **Pan-serotypic rRT-PCR C_T_** | **EA - O rRT-PCR C_T_** | **EA-A rRT-PCR C_T_** | **EA-SAT 1 rRT-PCR C_T_** | **EA-SAT 2 rRT-PCR C_T_** |
| --- | --- | --- | --- | --- | --- | --- | --- | --- | --- |
| 8177 | 26/05/2012 | Milk | Nyamburi |  | No C_T_ | NP | NP | NP | NP |
| 8146 | 30/07/2012 | Milk | Nyamburi |  | No C_T_ | NP | NP | NP | NP |
| 8233 | 17/08/2012 | Milk | Nyamburi |  | No C_T_ | NP | NP | NP | NP |
| 8233 | 17/08/2012 | Milk | Nyamburi |  | No C_T_ | NP | NP | NP | NP |
| 8401 | 18/08/2012 | Milk | Nyamsingisi |  | 24.93 | No C_T_ | 24.10 | No C_T_ | No C_T_ |
| 8266 | 17/10/2012 | Milk | Nyamburi |  | No C_T_ | NP | NP | NP | NP |
| 8233 | 18/10/2012 | Milk | Nyamburi |  | 36.46 | No C_T_ | No C_T_ | No C_T_ | No C_T_ |
| 8259 | 18/10/2012 | Milk | Nyamburi |  | No C_T_ | NP | NP | NP | NP |
| 8269 | 18/10/2012 | Milk | Nyamburi |  | No C_T_ | NP | NP | NP | NP |
| 8401 | 10/11/2012 | Milk | Nyamsingisi |  | No C_T_ | NP | NP | NP | NP |
| 8403 | 10/11/2012 | Milk | Nyamsingisi |  | No C_T_ | NP | NP | NP | NP |
| 8406 | 10/11/2012 | Milk | Nyamsingisi |  | No C_T_ | NP | NP | NP | NP |
| 8427 | 13/11/2012 | Milk | Nyichoka |  | No C_T_ | NP | NP | NP | NP |
| 8438 | 13/11/2012 | Milk | Nyichoka |  | No C_T_ | NP | NP | NP | NP |
| 8445 | 13/11/2012 | Milk | Nyichoka |  | No C_T_ | NP | NP | NP | NP |
| 8457 | 13/11/2012 | Milk | Nyichoka |  | No C_T_ | NP | NP | NP | NP |
| 7470 | 23/11/2012 | Milk | Rwamchanga |  | No C_T_ | NP | NP | NP | NP |
| 7476 | 23/11/2012 | Milk | Rwamchanga |  | No C_T_ | NP | NP | NP | NP |
| 7689 | 23/11/2012 | Milk | N/A |  | No C_T_ | NP | NP | NP | NP |
| 7652 | 24/11/2012 | Milk | Rwamchanga |  | No C_T_ | NP | NP | NP | NP |
| 7653 | 24/11/2012 | Milk | Rwamchanga |  | No C_T_ | NP | NP | NP | NP |
| 7655 | 24/11/2012 | Milk | Rwamchanga |  | No C_T_ | NP | NP | NP | NP |
| 7910 | 20/12/2012 | Milk | Tamau |  | No C_T_ | NP | NP | NP | NP |
| 7913 | 20/12/2012 | Milk | Tamau |  | No C_T_ | NP | NP | NP | NP |
| 7930 | 18/01/2013 | Milk | Mbilikili |  | 31.33 | No C_T_ | 32.73 | No C_T_ | No C_T_ |
| 7941 | 18/01/2013 | Milk | Mbilikili |  | No C_T_ | NP | NP | NP | NP |
| 7945 | 18/01/2013 | Milk | Mbilikili |  | 35.07 | No C_T_ | 35.56 | No C_T_ | No C_T_ |
| 7950 | 18/01/2013 | Milk | Mbilikili |  | 34.60 | No C_T_ | No C_T_ | No C_T_ | No C_T_ |
| 7951 | 18/01/2013 | Milk | Mbilikili |  | 26.99 | No C_T_ | 27.17 | No C_T_ | No C_T_ |
| 7963 | 18/01/2013 | Milk | Mbilikili |  | 29.58 | No C_T_ | 28.75 | No C_T_ | No C_T_ |
| 7964 | 18/01/2013 | Milk | Mbilikili |  | 25.93 | No C_T_ | 26.76 | No C_T_ | No C_T_ |
| 8110 | 04/02/2013 | Milk | Natambiso |  | No C_T_ | NP | NP | NP | NP |
| 8225 | 14/02/2013 | Milk | Motukeri |  | No C_T_ | NP | NP | NP | NP |
| 8404 | 15/02/2013 | Milk | Nyamsingisi |  | No C_T_ | NP | NP | NP | NP |
| 8406 | 15/02/2013 | Milk | Nyamsingisi |  | No C_T_ | NP | NP | NP | NP |
| 6605 | 11/11/2013 | Milk | Nygoti |  | No C_T_ | NP | NP | NP | NP |
| 9151 | 11/11/2013 | Milk | Nygoti |  | No C_T_ | NP | NP | NP | NP |
| 9152 | 11/11/2013 | Milk | Nygoti |  | No C_T_ | NP | NP | NP | NP |
| 6778 | 16/11/2013 | Milk | Nyamburi |  | No C_T_ | NP | NP | NP | NP |
| 8261 | 16/11/2013 | Milk | Nyamburi |  | No C_T_ | NP | NP | NP | NP |
| 8562 | 16/11/2013 | Milk | Nyamburi |  | 36.57 | No C_T_ | No C_T_ | 48.00 | No C_T_ |
| 8806 | 16/11/2013 | Milk | Nyamburi |  | No C_T_ | NP | NP | NP | NP |
| 8808 | 16/11/2013 | Milk | N/A |  | No C_T_ | NP | NP | NP | NP |
| 8809 | 16/11/2013 | Milk | N/A |  | 36.72 | No C_T_ | No C_T_ | No C_T_ | No C_T_ |
| 8811 | 16/11/2013 | Milk | N/A |  | No C_T_ | NP | NP | NP | NP |
| 8149 | 18/11/2013 | Milk | Nyamburi |  | 32.39 | 41.40 | No C_T_ | 34.26 | No C_T_ |
| 8413 | 18/11/2013 | Milk | Nyamburi |  | No C_T_ | NP | NP | NP | NP |
| 8194 | 22/11/2013 | Milk | Motukeri |  | No C_T_ | NP | NP | NP | NP |
| 8193 | 25/11/2013 | Milk | Motukeri |  | 37.96 | No C_T_ | No C_T_ | No C_T_ | No C_T_ |
| 8530 | 25/11/2013 | Milk | Mbilikili |  | No C_T_ | NP | NP | NP | NP |
| 9202 | 19/01/2014 | Milk | N/A |  | 33.53 | No C_T_ | No C_T_ | No C_T_ | No C_T_ |
| 7024 | 26/01/2014 | Milk | Nyichoka |  | No C_T_ | NP | NP | NP | NP |
| 9232 | 29/01/2014 | Milk | N/A |  | 33.67 | No C_T_ | No C_T_ | No C_T_ | No C_T_ |
| 6532 | 05/02/2014 | Milk | Tamau |  | No C_T_ | NP | NP | NP | NP |
| 6537 | 05/02/2014 | Milk | Tamau |  | No C_T_ | NP | NP | NP | NP |
| 7700 | 05/02/2014 | Milk | Tamau |  | No C_T_ | NP | NP | NP | NP |
| 8850 | 05/02/2014 | Milk | N/A |  | No C_T_ | NP | NP | NP | NP |
| 6545 | 06/02/2014 | Milk | Tamau |  | No C_T_ | NP | NP | NP | NP |
| 6551 | 06/02/2014 | Milk | Tamau |  | No C_T_ | NP | NP | NP | NP |
| 6582 | 06/02/2014 | Milk | Tamau |  | No C_T_ | NP | NP | NP | NP |
| 7908 | 06/02/2014 | Milk | Tamau |  | No C_T_ | NP | NP | NP | NP |
| 7909 | 06/02/2014 | Milk | Tamau |  | 35.48 | No C_T_ | No C_T_ | No C_T_ | No C_T_ |
| 7913 | 06/02/2014 | Milk | Tamau |  | 32.41 | 38.05 | No C_T_ | 35.65 | No C_T_ |
| 7914 | 06/02/2014 | Milk | Tamau |  | No C_T_ | NP | NP | NP | NP |
| 7545 | 10/02/2014 | Milk | Mbilikili |  | No C_T_ | NP | NP | NP | NP |
| 8544 | 10/02/2014 | Milk | Mbilikili |  | 31.97 | 34.16 | No C_T_ | 36.25 | No C_T_ |
| 8687 | 10/02/2014 | Milk | N/A |  | 36.97 | No C_T_ | No C_T_ | 40.75 | No C_T_ |
| 7950 | 12/02/2014 | Milk | Mbilikili |  | 37.04 | No C_T_ | No C_T_ | 38.85 | No C_T_ |
| 7955 | 12/02/2014 | Milk | Mbilikili |  | 36.18 | No C_T_ | No C_T_ | 36.86 | No C_T_ |
| 7956 | 12/02/2014 | Milk | Mbilikili |  | No C_T_ | NP | NP | NP | NP |
| 7961 | 12/02/2014 | Milk | Mbilikili |  | 35.44 | No C_T_ | No C_T_ | 40.69 | No C_T_ |
| 7963 | 12/02/2014 | Milk | Mbilikili |  | No C_T_ | NP | NP | NP | NP |
| 8682 | 12/02/2014 | Milk | N/A |  | No C_T_ | NP | NP | NP | NP |
| 8884 | 12/02/2014 | Milk | N/A |  | No C_T_ | NP | NP | NP | NP |
| 8889 | 12/02/2014 | Milk | N/A |  | No C_T_ | NP | NP | NP | NP |
| 8897 | 12/02/2014 | Milk | N/A |  | No C_T_ | NP | NP | NP | NP |
| 8110 | 18/02/2014 | Milk | Natambiso |  | 36.83 | No C_T_ | No C_T_ | No C_T_ | No C_T_ |
| 8300 | 19/02/2014 | Milk | Nyamsingisi |  | No C_T_ | NP | NP | NP | NP |
| 8401 | 19/02/2014 | Milk | Nyamsingisi |  | No C_T_ | NP | NP | NP | NP |
| 8403 | 19/02/2014 | Milk | Nyamsingisi |  | No C_T_ | NP | NP | NP | NP |
| 8533 | 19/02/2014 | Milk | Nyamsingisi |  | 39.66 | No C_T_ | No C_T_ | No C_T_ | No C_T_ |
| 8534 | 19/02/2014 | Milk | Nyamsingisi |  | No C_T_ | NP | NP | NP | NP |
| 9200 | 19/02/2014 | Milk | N/A |  | 34.66 | 38.01 | No C_T_ | 37.20 | No C_T_ |
| 9201 | 19/02/2014 | Milk | N/A |  | 35.38 | No C_T_ | No C_T_ | 36.38 | No C_T_ |
| 9203 | 19/02/2014 | Milk | N/A |  | No C_T_ | NP | NP | NP | NP |
| 9204 | 19/02/2014 | Milk | N/A |  | No C_T_ | NP | NP | NP | NP |
| 9205 | 19/02/2014 | Milk | N/A |  | No C_T_ | NP | NP | NP | NP |
| 9206 | 19/02/2014 | Milk | N/A |  | No C_T_ | NP | NP | NP | NP |
| 7459 | 22/02/2014 | Milk | Rwamchanga |  | 37.69 | No C_T_ | No C_T_ | No C_T_ | No C_T_ |
| 7461 | 22/02/2014 | Milk | Rwamchanga |  | 35.68 | No C_T_ | No C_T_ | No C_T_ | No C_T_ |
| 7472 | 22/02/2014 | Milk | Rwamchanga |  | 33.69 | No C_T_ | No C_T_ | No C_T_ | No C_T_ |
| 7476 | 22/02/2014 | Milk | Rwamchanga |  | No C_T_ | NP | NP | NP | NP |
| 7479 | 22/02/2014 | Milk | Rwamchanga |  | No C_T_ | NP | NP | NP | NP |
| 7485 | 22/02/2014 | Milk | Rwamchanga |  | No C_T_ | NP | NP | NP | NP |
| 7487 | 22/02/2014 | Milk | Rwamchanga |  | No C_T_ | NP | NP | NP | NP |
| 7494 | 22/02/2014 | Milk | Rwamchanga |  | No C_T_ | NP | NP | NP | NP |
| 7652 | 22/02/2014 | Milk | Rwamchanga |  | No C_T_ | NP | NP | NP | NP |
| 7653 | 22/02/2014 | Milk | Rwamchanga |  | No C_T_ | NP | NP | NP | NP |
| 7665 | 22/02/2014 | Milk | Rwamchanga |  | No C_T_ | NP | NP | NP | NP |
| 7670 | 22/02/2014 | Milk | Rwamchanga |  | No C_T_ | NP | NP | NP | NP |
| 8694 | 22/02/2014 | Milk | Tamau |  | No C_T_ | NP | NP | NP | NP |
| 8698 | 22/02/2014 | Milk | Rwamchanga |  | No C_T_ | NP | NP | NP | NP |
| 7050 | 24/02/2014 | Milk | Nyichoka |  | No C_T_ | NP | NP | NP | NP |
| 9310 | 24/02/2014 | Milk | N/A |  | No C_T_ | NP | NP | NP | NP |
| 9311 | 24/02/2014 | Milk | Nyichoka |  | No C_T_ | NP | NP | NP | NP |
| 9312 | 24/02/2014 | Milk | N/A |  | 37.41 | No C_T_ | No C_T_ | No C_T_ | No C_T_ |
| 9313 | 24/02/2014 | Milk | N/A |  | No C_T_ | NP | NP | NP | NP |
| 9315 | 24/02/2014 | Milk | N/A |  | No C_T_ | NP | NP | NP | NP |
| 7028 | 26/02/2014 | Milk | Nyichoka |  | No C_T_ | NP | NP | NP | NP |
| 7030 | 26/02/2014 | Milk | Nyichoka |  | 35.61 | 40.80 | No C_T_ | No C_T_ | No C_T_ |
| 7033 | 26/02/2014 | Milk | Nyichoka |  | 35.58 | No C_T_ | No C_T_ | No C_T_ | No C_T_ |
| 7040 | 26/02/2014 | Milk | Nyichoka |  | 37.18 | 40.35 | No C_T_ | No C_T_ | No C_T_ |
| 7043 | 26/02/2014 | Milk | Nyichoka |  | No C_T_ | NP | NP | NP | NP |
| 7048 | 26/02/2014 | Milk | Nyichoka |  | No C_T_ | NP | NP | NP | NP |
| 6625 | 28/03/2014 | Milk | Natambiso |  | No C_T_ | NP | NP | NP | NP |
| 6644 | 28/03/2014 | Milk | Natambiso |  | No C_T_ | NP | NP | NP | NP |
| 6657 | 28/03/2014 | Milk | Natambiso |  | No C_T_ | NP | NP | NP | NP |
| 6585 | 14/04/2014 | Milk | Nygoti |  | No C_T_ | NP | NP | NP | NP |
| 6599 | 14/04/2014 | Milk | Nygoti |  | 35.06 | No C_T_ | No C_T_ | 39.79 | No C_T_ |
| 6605 | 14/04/2014 | Milk | Nygoti |  | No C_T_ | NP | NP | NP | NP |
| 9151 | 14/04/2014 | Milk | Nygoti |  | 36.80 | 37.36 | No C_T_ | 35.96 | No C_T_ |
| 9152 | 14/04/2014 | Milk | Nygoti |  | 36.71 | No C_T_ | No C_T_ | No C_T_ | No C_T_ |
| 6675 | 19/05/2014 | Milk | Nygoti |  | No C_T_ | NP | NP | NP | NP |
| 6700 | 19/05/2014 | Milk | Nygoti |  | 36.64 | No C_T_ | No C_T_ | 39.71 | No C_T_ |
| 9243 | 20/05/2014 | Milk | N/A |  | 38.58 | No C_T_ | No C_T_ | No C_T_ | No C_T_ |
| 9247 | 20/05/2014 | Milk | N/A |  | No C_T_ | NP | NP | NP | NP |
| 9248 | 20/05/2014 | Milk | N/A |  | No C_T_ | NP | NP | NP | NP |
| 8257 | 23/05/2014 | Milk | Nyamburi |  | No C_T_ | NP | NP | NP | NP |
| 8565 | 23/05/2014 | Milk | Nyamburi |  | 35.48 | No C_T_ | No C_T_ | No C_T_ | No C_T_ |
| 8806 | 23/05/2014 | Milk | Nyamburi |  | No C_T_ | NP | NP | NP | NP |
| 8809 | 23/05/2014 | Milk | N/A |  | 24.54 | 26.77 | 31.33 | 26.52 | No C_T_ |
| 8177 | 26/05/2014 | Milk | Nyamburi |  | No C_T_ | NP | NP | NP | NP |
| 8413 | 26/05/2014 | Milk | Nyamburi |  | No C_T_ | NP | NP | NP | NP |
| 8816 | 26/05/2014 | Milk | Nyamburi |  | 37.09 | No C_T_ | No C_T_ | No C_T_ | No C_T_ |
| 8829 | 26/05/2014 | Milk | N/A |  | No CT | NP | NP | NP | NP |
| 8643 | 30/05/2014 | Milk | Nygoti |  | 34.60 | No C_T_ | No C_T_ | No C_T_ | No C_T_ |
| 8646 | 30/05/2014 | Milk | Nygoti |  | 35.53 | No C_T_ | No C_T_ | 39.09 | No C_T_ |
| 9149 | 30/05/2014 | Milk | Nygoti |  | No C_T_ | NP | NP | NP | NP |
| 8225 | 18/08/2014 | Milk | Motukeri |  | 32.90 | 37.00 | No C_T_ | 37.01 | No C_T_ |
| 9111 | 18/08/2014 | Milk | Motukeri |  | 36.38 | 37.25 | No C_T_ | 48.18 | No C_T_ |
| 7736 | 03/09/2014 | Milk | Nyichoka |  | 33.31 | No C_T_ | No C_T_ | No C_T_ | No C_T_ |
| 7601 | 09/09/2014 | Milk | N/A |  | 23.55 | No C_T_ | No C_T_ | 23.79 | No C_T_ |
| 7602 | 09/09/2014 | Milk | N/A |  | 31.01 | No C_T_ | No C_T_ | 31.27 | No C_T_ |
| 7608 | 09/09/2014 | Milk | N/A |  | 23.28 | 34.92 | No C_T_ | 31.76 | No C_T_ |
| 7609 | 09/09/2014 | Milk | Nyichoka |  | 30.79 | 33.11 | 37.07 | 32.43 | No C_T_ |
| 7716 | 29/09/2014 | Milk | N/A |  | No C_T_ | NP | NP | NP | NP |
| 7730 | 08/10/2014 | Milk | N/A |  | No C_T_ | NP | NP | NP | NP |
| 7743 | 08/10/2014 | Milk | N/A |  | No C_T_ | NP | NP | NP | NP |
| 7805 | 09/10/2014 | Milk | Bunchugu |  | 27.39 | No C_T_ | No C_T_ | 27.05 | No C_T_ |
| 7808 | 10/10/2014 | Milk | N/A |  | No C_T_ | NP | NP | NP | NP |
| 7815 | 16/10/2014 | Milk | Rwamchanga |  | 26.30 | No C_T_ | No C_T_ | 24.48 | No C_T_ |
| 7828 | 16/10/2014 | Milk | N/A |  | 37.75 | No C_T_ | No C_T_ | No C_T_ | No C_T_ |
| 7832 | 16/10/2014 | Milk |  |  | 35.66 | No C_T_ | No C_T_ | No C_T_ | No C_T_ |
| 7834 | 16/10/2014 | Milk | N/A |  | No C_T_ | NP | NP | NP | NP |
| 7848 | 16/10/2014 | Milk |  |  | 32.76 | 37.76 | No C_T_ | 40.70 | No C_T_ |
| 8011 | 07/11/2014 | Milk | N/A |  | 29.62 | No C_T_ | No C_T_ | 32.62 | No C_T_ |
| 8014 | 07/11/2014 | Milk | N/A |  | No C_T_ | NP | NP | NP | NP |
| 8021 | 07/11/2014 | Milk | N/A |  | 33.81 | No C_T_ | No C_T_ | No C_T_ | No C_T_ |
| 8032 | 07/11/2014 | Milk | N/A |  | 34.20 | 47.61 | No C_T_ | 37.21 | No C_T_ |
| 8039 | 07/11/2014 | Milk | N/A |  | No C_T_ | NP | NP | NP | NP |
| 8040 | 07/11/2014 | Milk | N/A |  | 33.70 | 37.24 | No C_T_ | 36.48 | No C_T_ |
| 8044 | 07/11/2014 | Milk | N/A |  | 35.13 | No C_T_ | No C_T_ | No C_T_ | No C_T_ |
| 8045 | 11/11/2014 | Milk | N/A |  | No C_T_ | NP | NP | NP | NP |
| 8227 | 25/11/2014 | Milk | Motukeri |  | No C_T_ | NP | NP | NP | NP |
| 7033 | 27/11/2014 | Milk | Nyichoka |  | No C_T_ | NP | NP | NP | NP |
| 9013 | 27/11/2014 | Milk | Nyichoka |  | 39.58 | No C_T_ | No C_T_ | No C_T_ | No C_T_ |
| 7960 | 02/12/2014 | Milk | Mbilikili |  | 31.62 | 35.85 | No C_T_ | 33.34 | No C_T_ |
| 7961 | 02/12/2014 | Milk | Mbilikili |  | 40.31 | No C_T_ | No C_T_ | No C_T_ | No C_T_ |
| 8840 | 02/12/2014 | Milk | Motukeri |  | No C_T_ | NP | NP | NP | NP |
| 8884 | 02/12/2014 | Milk | N/A |  | 31.78 | 35.06 | No C_T_ | 44.97 | No C_T_ |
| 8502 | 18/12/2014 | Milk | Mbilikili |  | 37.69 | No C_T_ | No C_T_ | No C_T_ | No C_T_ |
| 8517 | 18/12/2014 | Milk | Mbilikili |  | 35.43 | No C_T_ | No C_T_ | No C_T_ | No C_T_ |
| 9511 | 18/12/2014 | Milk | N/A |  | 32.56 | 36.07 | No C_T_ | 33.94 | No C_T_ |
| 8644 | 26/12/2014 | Milk | Nygoti |  | No C_T_ | NP | NP | NP | NP |
| 9205 | 09/01/2015 | Milk | N/A |  | 36.99 | No C_T_ | No C_T_ | No C_T_ | No C_T_ |
| 9144 | 28/01/2015 | Milk | Nyichoka |  | 30.14 | 34.54 | 37.05 | 37.40 | No C_T_ |
| 9150 | 28/01/2015 | Milk | Nygoti |  | No C_T_ | NP | NP | NP | NP |
| 9201 | 28/01/2015 | Milk | N/A |  | 27.37 | 36.80 | No C_T_ | 34.45 | No C_T_ |
| 9202 | 28/01/2015 | Milk | N/A |  | 36.33 | No C_T_ | No C_T_ | No C_T_ | No C_T_ |
| 6778 | 29/01/2015 | Milk | Nyamburi |  | No C_T_ | NP | NP | NP | NP |
| 6779 | 29/01/2015 | Milk | Nyamburi |  | No C_T_ | NP | NP | NP | NP |
| 8261 | 29/01/2015 | Milk | Nyamburi |  | No C_T_ | NP | NP | NP | NP |
| 8806 | 29/01/2015 | Milk | Nyamburi |  | No C_T_ | NP | NP | NP | NP |
| 8808 | 29/01/2015 | Milk | N/A |  | No C_T_ | NP | NP | NP | NP |
| 8811 | 29/01/2015 | Milk | N/A |  | 34.58 | No C_T_ | No C_T_ | No C_T_ | No C_T_ |
| 9227 | 29/01/2015 | Milk | N/A |  | No C_T_ | NP | NP | NP | NP |
| 7605 | 13/03/2015 | Milk | N/A |  | No C_T_ | NP | NP | NP | NP |
| 7951 | 02/12/2015 | Milk | N/A |  | 32.30 | 35.31 | No C_T_ | No C_T_ | No C_T_ |
| 6516 | N/A | Milk | Tamau |  | 32.27 | No C_T_ | No C_T_ | 37.89 | No C_T_ |
| 8297 | N/A | Milk | Nyamsingisi |  | No C_T_ | NP | NP | NP | NP |
| TAN/19/2012 (SAT 2) | 28/04/2012 | Cell culture isolate | Simanjiro |  |  |  |  |  |  |
| TAN/39/2012 (O) | 31/05/2012 | Cell culture isolate | Ngorongoro district |  |  |  |  |  |  |
| TAN/6/2013 (A) | 16/03/2013 | Cell culture isolate | Nyamburi |  |  |  |  |  |  |
| TAN/33/2014 (SAT 1) | 16/10/2014 | Cell culture isolate | Rwamchanga |  |  |  |  |  |  |
| TAN/20/2014 | 03/09/2014 | Epithelium | Nyichoka |  |  |  |  |  |  |
| TAN/22/2014 | 09/09/2014 | Epithelium | Nyichoka |  |  |  |  |  |  |
| TAN/23/2014 | 09/09/2014 | Vesicular fluid | Nyichoka |  |  |  |  |  |  |
| TAN/28/2014 | 09/10/2014 | Epithelium | Bunchugu |  |  |  |  |  |  |
| TAN/29/2014 | 09/10/2014 | Vesicular fluid | Bunchugu |  |  |  |  |  |  |
| TAN/34/2014 | 16/10/2014 | Epithelium | Rwamchanga |  |  |  |  |  |  |

N/A – information not available. NP – Not performed. No C_T_ – No C_T_ value observed (>50).
